# Inappropriate Underdosing of Direct Oral Anticoagulants in Atrial Fibrillation Patients: Results from the START2-AF Registry

**DOI:** 10.3390/jcm13072009

**Published:** 2024-03-29

**Authors:** Daniela Poli, Emilia Antonucci, Walter Ageno, Martina Berteotti, Anna Falanga, Vittorio Pengo, Paolo Chiarugi, Benilde Cosmi, Carmelo Paparo, Antonio Chistolini, Antonio Insana, Domenico Lione, Giuseppe Malcangi, Giuliana Martini, Lucilla Masciocco, Simona Pedrini, Eugenio Bucherini, Daniele Pastori, Pasquale Pignatelli, Andrea Toma, Sophie Testa, Gualtiero Palareti, START AF Study Group

**Affiliations:** 1 Center of Atherothrombotic Disease, Azienda Ospedaliero-Universitaria Careggi, 50134 Firenze, Italy; martina.berteotti@unifi.it; 2 Fondazione Arianna Anticoagulazione, 40138 Bologna, Italy; e.antonucci@fondazionearianna.org (E.A.); gualtiero.palareti@unibo.it (G.P.); 3 SSD Degenza Breve Internistica, Dipartimento di Medicina Interna, Ospedale di Circolo e Fondazione Macchi, ASST Sette Laghi, 21100 Varese, Italy; walter.ageno@uninsubria.it; 4 School of Medicine, Università di Milano Bicocca, 20126 Milano, Italy; annafalanga@yahoo.com; 5 Immunoematologia e Medicina Trasfusionale ASST Papa Giovanni XXIIIo, 24127 Bergamo, Italy; 6 Dipartimento di Scienze Cardio-Toraco-Vascolari, AOU Padova, 35121 Padova, Italy; vittorio.pengo@unipd.it; 7 UO di Analisi Chimico Cliniche, Azienda Ospedaliero Universitaria Pisana, 56100 Pisa, Italy; p.chiarugi@ao-pisa.toscana.it; 8 Division of Angiology and Blood Coagulation, IRCCS Azienda Ospedaliero-Universitaria S. Orsola-Malpighi Bologna, 40138 Bologna, Italy; benilde.cosmi@unibo.it; 9 Laboratorio Analisi, Ospedale Maggiore, 10023 Chieri, Italy; carmelo.paparo@libero.it; 10 Dipartimento di Medicina Traslazionale e di Precisione, Sapienza Università di Roma, 00185 Roma, Italy; antonio.chistolini@uniroma1.it; 11 SC Laboratorio Analisi Chimico-Cliniche e Microbiologia, A.O. Ordine Mauriziano, 10128 Torino, Italy; ainsana@mauriziano.it; 12 UOC di Patologia Clinica, Ospedale A Perrino, 72100 Brindisi, Italy; dr.lione@libero.it; 13 U.O. Medicina Trasfusionale, Azienda Ospedaliero-Universitaria Policlinico di Bari, 70124 Bari, Italy; giuseppe.malcangi@policlinico.ba.it; 14 Centro Emostasi, Spedali Civili Di Brescia, 25123 Brescia, Italy; giuliana.martini@asst-spedalicivili.it; 15 UOC Medicina Interna, Ospedale Lastaria, 71036 Lucera, Italy; lucillamasciocco@alice.it; 16 Servizio di Laboratorio, Istituto Ospedaliero Fondazione Poliambulanza, 25124 Brescia, Italy; simona.pedrini@poliambulanza.it; 17 Medicina Interna, Ambulatorio Emostasi Trombosi, Faenza (Ra) AUSL Romagna, 48018 Faenza, Italy; bucherini@gmail.com; 18 Emergency Medicine Unit, Department of Clinical, Internal, Anesthesiological and Cardiovascular Sciences, Sapienza University of Rome, 00185 Roma, Italy; daniele.pastori@uniroma1.it (D.P.); pasquale.pignatelli@uniroma1.it (P.P.); 19 UOC di Patologia Clinica, Ambulatorio Terapia Anticoagulante Orale, O.C. “L. Cazzavillan”, 36071 Arzignano, Italy; andrea.toma@aulss8.veneto.it; 20 Haemostasis and Thrombosis Centre, Laboratory Medicine Department, ASST Cremona, 26100 Cremona, Italy; sophie.testa@assst-cremona.it

**Keywords:** non valvular atrial fibrillation, bleeding, DOAC, low dosage, stroke

## Abstract

**Background:** Direct oral anticoagulants (DOACs) are recommended for stroke prevention in non-valvular atrial fibrillation (NVAF) patients. We aimed to describe the prevalence of inappropriate DOACs dose prescription in the START2-AF Registry, the outcomes according to the appropriateness of the dosage, and the factors associated with inappropriate dose prescription. **Methods:** Patients’ demographics and clinical data were prospectively collected as electronic files in an anonymous form on the website of the START2-Registry; DOACs dosage was determined to be appropriate when prescribed according to the European Heart Rhythm Association Guidelines. **Results:** We included 5943 NVAF patients on DOACs; 2572 (46.3%) were female patients. The standard dose (SD) was prescribed to 56.9% of patients and the low dose (LD) was prescribed to 43.1% of patients; 38.9% of all NVAF patients received an inappropriate LD DOAC and 0.3% received inappropriate SD. Patients treated with LD DOAC had a significantly higher rate of all bleedings (RR 1.5; 95% CI 1.2–2.0), major bleedings (RR 1.8; 95% CI 1.3–1.7), and mortality (RR 2.8; 95% CI 1.9–4.1) with respect to patients treated with SD DOAC. No difference was found among patients treated with appropriate and inappropriate LD regarding bleeding, thrombotic, and mortality rates. Age, body weight <60 kg, and renal failure were significantly associated with inappropriate LD DOAC prescription. **Conclusions:** Inappropriate LD DOACs in NVAF patients is not associated with a reduction in bleeding risk, nor with an increased thrombotic risk. Instead, it is associated with higher mortality rate, suggesting that, in clinical practice, underdosing is preferred for patients at particularly high risk for adverse events.

## 1. Introduction

Oral anticoagulation is the evidence-based practice for stroke risk reduction in patients with non-valvular atrial fibrillation (NVAF). The introduction of direct oral anticoagulants (DOACs) changed the landscape of anticoagulation and DOACs have become the first-line agents for stroke prevention in NVAF. All the four marketed DOACs are available in two dosages, a standard dose (SD) and a lower dose (LD), each of them indicated for patients with specific clinical characteristics, such as age, baseline renal function, weight, and concomitant medications, which potentially interact with the metabolism of these drugs [1,2,3,4,5]. The recommended dose for each DOAC is reported by the summary of product characteristics (SmPCs) and approved by the European Medicines Agency.

Several international scientific societies have produced practical guidelines to help clinicians in choosing the adequate dosage in clinical practice; in particular, the European Heart Rhythm Association (EHRA) published a practical guide on the optimal use of DOACs and of their different dosages [6]. However, clinicians often prescribe LD DOACs to patients who do not meet dose-reduction criteria, especially in older adults with multiple co-morbidities and who are at a high risk for bleeding [7,8,9,10].

In the GARFIELD-AF study, 23.2% of patients received an unrecommended LD DOAC, and 3.8% were overdosed [7]. High rates of underdosing, over 30% of patients, was observed in Latin American countries and in Asia [11]. In North America and in Europe, less than <20% of patients usually receive a low dosage [12], except in France, Germany, and Spain, where a rate of underdosage is reported in 20% to 30% of patients. On the contrary, overdosage is rarely observed and is reported to be less than 5% [7].

The aims of the present study were as follows: (1) to describe the prevalence of inappropriate DOACs dosage prescription in patients enrolled in the Italian START-AF Register; (2) to determine the occurrence of adverse thrombotic and hemorrhagic events and mortality in patients according to dosage and appropriateness of dosage prescription; (3) to investigate the factors associated with inappropriate dosage prescriptions.

## 2. Materials and Methods

The START-Register is an observational, multicenter, prospective cohort study that includes patients over 18 years old who have started anticoagulation therapy, regardless of the drug or the indication for treatment. The START-Register aims to collect data on the incidence of adverse events in patients taking anticoagulants, as well as their determinants. The study design and protocol were approved by the Ethical Committee of the S. Orsola-Malpighi University Polyclinic Hospital (Bologna, Italy) on 10 December 2011 (N.142/2010/0/0ss) (NCT02219984). The START-Register procedures and data collection have been previously described [13].

The present sub-study is focused on patients on treatment with DOACs enrolled in the START-Register, both naive and patients shifted from VKAs treatment.

Creatinine clearance (CrCl) was calculated by the Cockroft–Gault formula [14]. Renal failure was defined by an estimated CrCl <30 mL/min. Patients with non-valvular AF were stratified for stroke risk according to the CHA2DS2VASc score [15], while baseline bleeding risk was evaluated by the HAS-BLED score [16]. Major bleeding and clinically relevant non-major bleeding (CRNMB) were defined according to the classification proposed by the International Society on Thrombosis and Hemostasis [17,18]. Stroke diagnosis requires the abrupt onset of focal neurological symptoms lasting at least 24 h and supported by congruent ischemic lesions at the imaging scan. Systemic embolism is defined by symptoms consistent with an acute loss of blood flow to a peripheral artery, which is supported by the objective evidence of embolism.

DOACs dosage was analyzed and defined appropriate when the dose was prescribed in accordance with the indication reported in the Guidelines of the European Heart Rhythm Association [6]. If the prescriptiondid not fulfillthe indicated criteria the dosage was defined “ inappropriate”.

## 3. Statistical Analysis

A descriptive analysis was performed. Continuous variables are expressed as median and interquartile range or as mean ± standard deviation. Categorical variables are expressed as frequencies and percentages. Differences between continuous values were assessed using the unpaired t-test, and categorical variables were compared using the Chi-square test or the Fisher exact test, as appropriate. The incidence of bleeding events, thrombotic events, and death was calculated by dividing the number of events by the person’s time at risk. The incidence rate ratio together with the 95% confidence interval was calculated; *p* < 0.05 was considered statistically significant. Univariate and multivariate analyses were performed to identify the most relevant factors associated with inappropriate dosage prescriptions. The data were analyzed with the use of SPSS software for Windows, V.26 (IBM, Armonk, NY, USA), and Stata V.14 statistical software package (StataCorp, College Station, TX, USA).

## 4. Results

The START-AF study included 11,078 NVAF patients, enrolled in 47 Italian centers, distributed all over the country; 5135 patients (46.4%) were on VKA treatment, whereas 5943 patients (53.6%) were on DOACs.

The median age of DOACs patients was 79 years and 2572 (46.3%) were female patients. Patients were followed for a median time of 1.3 years (range 0.1–8.9 years); 4022 (67.7%) patients were naive to anticoagulation, and 1921 (32.3%) were first treated with warfarin, then switched to DOACs. Among patients on DOACs, 2562 (43.1%) were treated with the LD of the four available DOACs. The characteristics of patients are reported in Table 1. The median ages of LD and SD DOAC patients were 83 and 75 years, respectively. Female patients comprised 55.6% of the LD group and 39.3% of the SD DOAC. Moderate renal failure was present in 66.8% and in 27.6% of LD and SD patients, respectively, and severe renal failure was present in 7.1% and 0.3% of LD and SD patients, respectively. Heart failure, coronary artery disease, dementia, anemia, history of previous bleeding, and frequent fall were more frequent among patients treated with LD compared to those treated with SD DOAC (Table 1).

Patients treated with SD DOAC numbered 3376 (56.9%) and the prescription was appropriate in 3367 (99.7%). Instead, patients treated with LD DOAC showed an inappropriate prescription in 988 cases (38.6%). In particular, patients on treatment with apixaban showed inappropriate prescription in 54.1% of cases, patients on dabigatran showed inappropriate prescription in 40.3% of cases, patients on rivaroxaban showed inappropriate prescription in 40.5% of cases, and patients on edoxaban showed inappropriate prescription in only 16.1% of cases (Table 2).

During follow-up, 249 bleeding events (rate 2.5 × 100 pt-yrs) were recorded; 121 (rate 1.2 × 100 pt-yrs) were major bleedings, 32 of which (rate 0.3 × 100 pt-yrs) were intracerebral bleedings; 47 patients had a thromboembolic event (rate 0.5 × 100 pt-yrs), and 142 patients died (rate 1.4 × 100 pt-yrs) (Table 3). Patients treated with LD DOAC regimen had a significantly higher rate of all bleeding events (RR 1.5; 95% CI 1.2–2.0), major bleeding (RR1.8; 95% CI 1.3–1.7), and mortality (RR 2.8; 95% CI 1.9–4.1), with respect to patients treated with SD DOAC (Table 3).

When we look at the occurrence of adverse events among patients on LD DOAC regimen, no difference was found between those treated with appropriate and inappropriate LD (Table 4).

The univariate analysis demonstrates that female sex, age, body weight < 60 kg, renal failure, CHA_2_DS_2_VASC, and HAS-BLED scores were significantly associated with an inappropriate LD DOAC prescription (Table 5). However, when a multivariate analysis was performed, only age, body weight < 60 kg, and renal failure were significantly associated with inappropriate LD prescription. Instead, the associated treatment with antiplatelet drugs and the history of previous bleeding events were significantly associated with appropriate LD prescription.

## 5. Discussion

Here, we present data of NVAF patients treated with DOACs included in the START-AF Register. In our cohort, patients on DOACs received a reduced dose in 43.1% of the cases. A high prevalence of a reduced dose prescription was previously reported [10,19], even though, in these studies, a percentage ranging from 16% and 22% was reported, which is lower than that found in our study. The use of reduced doses of DOACs is also more frequent in routine clinical practice than in clinical trials [20,21,22]. Moreover, when reduced doses were prescribed, frequent inappropriate prescription has been reported, and some studies found an increased risk of thrombotic complications associated with inadequate anticoagulation. The elevated median age of our cohort may have contributed to the high prescription of a reduced dose, as it is known that older age is associated with anticoagulant underdosing. In our cohort, patients treated with LD had a median age of 83 years (IQR 79–87 years), which is 5 years older than the median age of patients treated with SD, and 55.6% of them were female patients. Patients included in the START-AF Register are older than patients included in the Garfield AF [23], ORBIT AF, and PREFER AF study [24]. In fact, 66.7% of patients were older than 75 years in our cohort, in comparison to less than 50% of patients included in the other studies. Instead, the mean CHA2DS2VASc score and the mean HAS-BLED score of our cohort were like those of the patients included in the quoted studies.

Looking at the appropriateness of dosage prescription according to the EHRA Guidelines, we found that 38.6% of patients treated with LD were considered to receive an inappropriate LD regimen. Instead, only 0.3% of patients received inappropriate SD regimen. In our cohort, the most-prescribed DOAC was apixaban (31.6% of cases). Inappropriate LD was more frequently found among patients on apixaban (54.1%); instead, it was found only in 16.1% of patients treated with edoxaban. Patients on dabigatran and rivaroxaban were treated with inappropriate LD in about 40% of cases. Patients with inappropriate LD were more frequently female patients, very elderly, with low body weight and renal failure.

However, when a multivariate analysis was performed, only age, low body weight and renal failure were confirmed to be significantly associated with inappropriate underdosing according to the high percentage of women among the very elderly patients and with the lower body weight of women with respect to men.

During follow-up, we recorded a significantly higher rate of all bleedings and of major bleedings among patients treated with the LD with respect to patients treated with SD. Similarly, patients treated with LD also showed a higher mortality rate (RR 2.8; 95% CI 1.9–4.1) with respect to patients treated with the SD. Instead, no difference was found in the rate of thrombotic events between the two groups.

A null effect of underdosing on the risk of stroke/TIA and of cardiovascular events has been reported in a recent metanalysis [25] of published studies, reporting data on inappropriate underdosing. Differently from our findings, a null effect was reported in that study for bleeding among patients treated with inappropriate LD; on the contrary, higher mortality rate was confirmed among patients treated with inappropriate LD [2], in keeping with our results. These results are surprising, because we reasonably expected to obtain a reduced bleeding risk and a high stroke risk when low-intensity anticoagulation is used. The confirmed null effect on the reduction in bleeding risk found in available studies, that is also higher in our data, and the null effect on the stroke risk that is not enhanced when less intense anticoagulation is used, raise a question about the indications on the optimal dosage of DOACs in NVAF patients. Should we reconsider the appropriateness of the guidelines for optimal dosage prescriptions? The trials that led to the marketing of DOACs were performed more than 10 years ago and were conducted on a population of NVAF patients who were different from those who are now followed in clinical practice. In the registration trials, the median age of the included patients varies from 70 to 72 years, almost a decade younger than the patients followed in our practice. The definition of ‘appropriate dosage’ was established based on these data, and it seems they do not fulfill the characteristics of the NVAF patients of the present.

Based on our results, it can be argued that clinicians fail to obtain a safety benefit in reducing bleeding risk when they use inappropriate underdosing. However, we may suppose that if an SD would have been used in these elderly patients, we could have recorded an increase in the bleeding risk. The elevated mortality rate of these patients seems to indicate that they are at a particularly high risk of adverse events. The ability of physicians to adequately tailor treatments for single patients probably goes beyond the rules defined by guidelines.

### Strengths and Limitations of the Study

The strengths of our study include the prospective design, the large cohort, and the participation of highly experienced centers. This study has also several limitations. Firstly, the study has an observational design and the results should be interpreted with caution against the risks of bias reported for these studies [26]. Secondly, the causes of death described in the electronic case report forms were reported as deaths related to bleeding events or to cerebral ischemic events. All the other causes of death were defined as being unrelated to anticoagulant treatment, and we cannot exclude an underestimation of adverse events. Moreover, in the electronic form, we collected the value of ejection fraction (EF); instead, data on episodes of heart failure were not always available. Therefore, we cannot exclude the possibility that we have missed some patients with heart failure with preserved EF. Finally, there was no central adjudication of the outcome events in this study.

In conclusion, our results confirmed that the use of appropriate or inappropriate low-dose DOACs in NVAF patients is not associated with a reduction in the bleeding risk; notwithstanding the lower dose of anticoagulant drugs, this was even higher among these patients. LD DOACs use is also not associated with an increased thrombotic risk, and it seems to be a therapeutic option to be reserved mainly for elderly patients who carry a particularly high risk for mortality. Further studies are needed to better understand optimal dosage of DOACs among very elderly patients.

## Figures and Tables

**Table 1:** Characteristics of patients.

	All Patients N (%)	Patients with SD (*) N (%)	Patients with LD (*) N (%)
Patients	5943 (53.6)	3376 (56.9)	2562 (43.1)
Female patients	2752 (46.3)	1328 (39.3)	1424 (55.6)
Male patients	3191 (53.7)	2048 (66.7)	1138 (44.4)
Median age ([IQR)	79 (72–84)	75 (69–80)	83 (79–87)
Age > 75 years	3966 (66.7)	1709 (50.7)	2253 (88.1)
Total follow-up (years)	10,120	5792	4313
Median follow-up (years) (range) [IQR]	1.3 (0.1–8.9)	1.3 (0.1–8.7)	1.2 (0.1–8.9)
	[1.0–2.0]	[1.0–2.0]	[1.0–2.0]
Paroxysmal AF	2340 (39.6)	1448 (43.0)	889 (34.9)
Hypertension	4887 (82.2)	2725 (80.7)	2158 (84.2)
Diabetes Mellitus	1226 (20.6)	710 (21.0)	516 (20.1)
Heart failure	1069 (18.0)	444 (13.2)	625 (24.4)
Coronary Artery Disease	848 (14.3)	438 (13.0)	410 (16.0)
Peripheral Occlusive Arterial Disease	316 (5.3)	172 (5.1)	144 (5.6)
COPD	732 (12.3)	367 (10.9)	365 (14.2)
Active Cancer	168 (2.8)	88 (2.6)	80 (3.1)
Dementia	228 (3.8)	75 (2.2)	153 (6.0)
Anemia	155 (2.6)	47 (1.4)	108 (4.2)
Platelet count < 100,000	43 (0.7)	18 (0.5)	25 (1.0)
Bed rest	66 (1.1)	21 (0.6)	45 (1.8)
Frequent fall	112 (1.9)	40 (1.2)	72 (2.8)
Previous bleeding	261 (4.4)	98 (2.9)	163 (6.4)
Previous TIA/Stroke	963 (16.2)	533 (15.8)	430 (16.7)
Renal function (5907/5943)			
eGFR < 30 mL/min	191 (3.2)	10 (0.3)	181 (7.1)
30–60 mL/min	2629 (44.2)	927 (27.6)	1702 (66.8)
Antiplatelet drugs	599 (10.1)	288 (8.5)	311 (12.1)
Psychotropic drugs	552 (9.3)	262 (7.8)	289 (11.3)
Mean CHA_2_DS_2_VASc score (SD)	3.7 (1.5)	3.4 (1.4)	4.2 (1.3)
CHA_2_DS_2_VASc ≤ 2	1119 (18.8)	912 (27.0)	207 (8.1)
Mean HAS-BLED score (SD)	2.1 (0.8)	2.0 (0.8)	2.3 (0.8)
HAS-BLED ≥ 3	1505 (25.3)	675 (20.0)	828 32.3
Anticoagulant Treatment			
Naïve	4022 (67.7)	2294 (68.0)	1723 (67.3)
Switched from VKA	1921 (32.3)	1082 (32.0)	839 (32.7)
Apixaban	1880 (31.6)	1157 (34.3)	723 (28.2)
Dabigatran	1397 (23.5)	627 (18.6)	765 (29.9)
Edoxaban	1101 (18.5)	553 (16.4)	548 (21.4)
Rivaroxaban	1565 (26.3)	1039 (30.8)	526 (20.5)
DOAC low dose	2562 (43.1)	-	2562

* Data not available for 5 patients.

**Table 2:** Distribution of patients according to the drug used and to appropriateness of dosage.

	N.	N. Patients with SD	N. Patients with Appropriate SD	N. Patients with Inappropriate SD	N. Patients with LD	N. Patients with Appropriate LD	N. Patients with Inappropriate LD (*)
apixaban	1880	1157 (61.5)	1148 (99.2)	9 (0.8)	723 (38.5)	332 (45.9)	380 (54.1)
dabigatran	1397	627 (45.0)	627 (100.0)	-	765 (55.0)	457 (59.7)	308 (40.3)
edoxaban	1101	553 (50.2)	553 (100.0)	-	548 (49.8)	457 (83.4)	88 (16.1)
rivaroxaban	1565	1039 (66.4)	1039 (100.0)	-	526 (33.6)	312 (59.5)	212 (40.5)
Total	5943	3376 (56.9)	3367 (99.7)	9 (0.3)	2562 (43.1)	1558 (60.8)	988 (38.6)

(*) Data not available for 18 patients; SD—standard dose; LD—low dose.

**Table 3:** Adverse events recorded during follow-up according to treatment with SD DOACs and LD DOAC.

	Total N. Events (Rate × 100 pt-yrs)	N. Events in SD (Rate × 100 pt-yrs)	N. of Events in LD (Rate × 100 pt-yrs)	RR (95%CI)	*p*
N. patients	5943	3376	2562		
PT-yrs	10,120	5792	4313		
All Bleedings	249 (2.5)	115 (2.0)	134 (3.1)	1.5 (1.2–2.0)	0.004
Major Bleedings	121 (1.2)	51 (0.9)	70 (1.6)	1.8 (1.3–2.7)	0.008
ICH	32 (0.3)	14 (0.2)	18 (0.4)	1.7(0.8–3.7)	0.1
GI bleed	37 (0.4)	17 (0.3)	20 (0.5)	1.6 (0.8–3.2)	0.7
CRNMB	128 (1.3)	64 (1.1)	64 (1.5)	1.3 (0.9–1.9)	1
Fatal bleedings	6 (0.06)	1 (0.02)	5 (0.1)	/	
Thrombotic events	86 (0.9)	42 (0.7)	44 (1.0)	1.4 (0.9–2.2)	0.1
Stroke/TIA/AE	47 (0.5)	24 (0.4)	23 (0.5)	1.3 (0.7–2.4)	0.3
Fatal Stroke	1 (0.01)	1 (0.02)	-	/	
Death	142 (1.4)	46 (0.8)	96 (2.2)	2.8 (1.9–4.1)	0.000

ICH—intracranial bleeding; GI—gastrointestinal bleeding; TIA—transient ischemic attack; AE—arterial embolism; SD—standard dose; LD—low dose.

**Table 4:** Adverse events recorded during follow-up according to appropriate or inappropriate LD DOACs.

	N. Events in Appropriate Low Dose	N. of Events in Inappropriate Low Dose	RR (95%CI)	*p*
N. of patients	1558	988		
Pt-years	2461	1820		
All Bleedings	76 (3.1)	57 (3.1)	1 (0.7–1.4)	0.9
Major Bleedings	43 (1.7)	26 (1.4)	1.2 (0.7–2.1)	0.4
ICH	11 (0.4)	7 (0.4)	1.1 (0.4–3.5)	0.8
GI bleed	10 (0.4)	10 (0.5)	0.7 (0.3–2.0)	0.5
CRNMB	33 (1.3)	31 (1.7)	0.8 (0.5–1.3)	0.3
Fatal bleedings	4 (0.02)	1 (0.05)	/	
Cardiovascular events	11 (0.4)	9 (0.5)	0.9 (0.3–2.4)	0.8
Stroke/TIA/AE	18 (0.7)	6 (0.3)	2.1 (0.8–6.8)	0.08
Fatal Stroke	1 (0.04)	1 (0.05)	/	
Death	96 (4.0)	54 (3.0)	1.3 (0.9–1.9)	0.1

**Table 5:** Univariate and multivariate analysis for risk factors associated with inappropriate LD DOAC prescription.

	Univariate Analysis OR (95% CI)	Multivariate Analysis OR (95% CI)
Female sex	2.0 (1.7–2.4)	0.9 (0.7–1.1)
Age	1.2 (1.1–1.2)	1.1 (1.1–1.1)
CHA2DS2VASC score	1.3 (1.2–1.4)	1.0 (0.9–1.2)
HAS-BLED score	1.2 (1.0–1.3)	1.1 (0.9–1.3)
Body weight < 60 Kg	6.6 (5.3–8.3)	5.9 (4.6–7.6)
Anemia	1.4 (0.9–2.1)	0.9 (0.6–1.6)
eGFR < 60 mL/min	6.2 (5.1–7.6)	2.6 (2.0–3.2)
Dementia	0.5 (0.3–0.7)	1.0 (0.7–1.6)
Heart failure	1.1 (0.9–1.3)	1.1 (0.8–1.4)
Associated antiplatelets	0.8 (0.6–1.0)	0.7 (0.5–1.0)
Previous bleeding	0.4 (0.3–0.5)	0.3 (0.2–0.5)

## Data Availability

The data presented in this study are available upon reasonable request from Fondazione Arianna Anticoagulazione.

## Appendix A

The list of participating centers and investigators who contributed to the START2 AF Registry is presented here:

- Benilde Cosmi, Division of Angiology and Blood Coagulation, IRCCS Azienda Ospedaliero-Universitaria S. Orsola-Malpighi Bologna, Italy.
- Daniela Poli, Elena Lotti, Martina Berteotti, Rossella Marcucci SOD Malattie Aterotrombotiche, Azienda Ospedaliero Universitaria-Careggi, Firenze, Italy.
- Walter Ageno, Giovanna Colombo, SSD Degenza Breve Internistica, Dipartimento di Medicina Interna, Ospedale di Circolo e Fondazione Macchi, ASST Sette Laghi, Varese, Italy.
- Doris Barcellona, UOC Medicina Interna, Centro Emocoagulopatie, AOU Cagliari, Cagliari.
- Salvatore Bradamante, Centro Trasfusionale, Centro Emostasi e Trombosi, Ospedale SS Annunziata, Taranto, Italy.
- Eugenio Bucherini, Luca Bucherini, Luca Ielasi, Maria Laura Lazzari, Medicina Interna-Ambulatorio Emostasi Trombosi, AUSL Romagna—Ravenna, Italy.
- Antonio Ciampa, Dipartimento Medicina di Laboratorio, Laboratorio Analisi, Ospedale San Giuseppe Moscati, Avellino, Italy.
- Antonio Chistolini, Alessandra Serrao, Dipartimento di Medicina Traslazionale e di Precisione Sapienza Università di Roma—Roma, Italy.
- Luciano Crippa Ambulatorio Emostasi e Trombosi, UO Patologie Trombotiche Ospedale San Raffaele, Milano, Italy.
- Raimondo De Cristofaro, Erica De Candia, UOSD Malattie Emorragiche e Trombotiche, Dipartimento Diagnostica per Immagini, Radioterapia Oncologica ed Ematologia, Fondazione Policlinico Universitario Agostino Gemelli IRCCS, Roma, Italy.
- Valeria De Micheli, Centro Emostasi e Trombosi, Medicina Generale, ASST Lecco, Lecco, Italy.
- Igor Diemberger, Giuseppe Boriani, Dipartimento Cardiotoraco Vascolare, IRCCS Azienda Ospedaliero-Universitaria S. Orsola-Malpighi Bologna, Italy.
- Marcello Di Nisio, Ettore Porreca Dipartimento di Medicina e scienze dell’invecchiamento, SSD Medicina Interna University “G.D’Annunzio” di Chieti-Pescara, Pescara Italy.
- Anna Falanga, Luca Barcella, Università Bicocca Milano, Dept. Medicine and Surgery, Monza and Divisione di Immunoematologia e Medicina Trasfusionale and Centro Emostasi e Trombosi, ASST Papa Giovanni XXIII—Bergamo, Italy.
- Antonio Insana, SC Laboratorio Analisi Chimico-Cliniche e Microbiologia. A.O. Ordine Mauriziano, Torino; Italy.
- Nicola Lucio Liberato, UO Medicina Interna, Ambulatorio Terapia Anticoagulante Orale, AO Provincia di Pavia, Pavia, Italy.
- Domenico Lione, UOC di Patologia Clinica, Ospedale A Perrino, Brindisi, Italy.
- Rosa Maria Lombardi, Centro Emostasi e Trombosi, UO Medicina Interna, Azienda Ospedaliero-Universitaria di Parma, Italy.
- Giacomo Lucarelli, Centro Trombosi, Ospedale Regionale F. Miulli, Acquaviva delle Fonti (Bari), Italy.
- Giuseppe Malcangi, Centro Emofilia e Trombosi, Policlinico di Bari, Bari, Italy.
- Catello Mangione, Trasfusionale, Ospedale di Galatina, Galatina (LE), Italy.
- Giuliana Martini Centro Emostasi, Spedali Civili Di Brescia, Brescia, Italy.
- Carmelo Paparo, Laboratorio Analisi, Ospedale Maggiore Chieri, Torino, Italy.
- Simona Pedrini, Franco Capuzzi, Servizio di Laboratorio, Istituto Ospedaliero Fondazione Poliambulanza, Brescia; Italy.
- Vittorio Pengo, Dipartimento di Scienze Cardio-Toraco-Vascolari, AOU Padova, Padova, Italy.
- Antonietta Piana, Francesco Cibecchini, Centro Prevenzione e Cura Malattie Tromboemboliche e Centro TAO, I.R.C.C.S. Azienda Ospedaliera Universitaria San Martino-IST, Genova, Italy.
- Pasquale Pignatelli, Daniele Pastori, Centro Trombosi, Dipartimento SCIAC, Sapienza Università di Roma, Italy.
- Vincenza Rossi, Centro Emostasi e Trombosi, UOC Ematologia, Azienda Ospedaliera di Cosenza, Cosenza Italy.
- Lucia Ruocco, Paolo Chiarugi, UO di Analisi Chimico Cliniche, Azienda Ospedaliero Universitaria Pisana, Pisa, Italy.
- Domizio Serra, Servizio Analisi, Ospedale Evangelico Internazionale, Sede di Castelletto, Genova, Italy.
- Carmine Spataro, Margherita Reduzzi, Chiara ambaglio SIMT ASST Bergamo Ovest—Ospedale di Treviglio, Pavia, Italy.
- Sophie Testa, Oriana Paoletti, Centro Emostasi e Trombosi, UUOO Laboratorio Analisi chimico-cliniche e microbiologiche, ASST Cremona, Cremona, Italy.
- Andrea Toma, UOC di Patologia Clinica, Ambulatorio Terapia Anticoagulante Orale, O.C. “L. Cazzavillan” Arzignano, (Vicenza), Italy.
- Rita Carlotta Santoro Centro Emostasi e Trombosi, UO Emofilia e Patologie della Coagulazione, Dipartimento di Ematologia, Oncologia e Medicina Trasfusionale, Azienda Ospedaliera “Pugliese Ciaccio”, Catanzaro, Italy.
- Claudio Vasselli, Laboratorio Patologia Clinica, Policlinico Casilino- Roma, Roma Italy.

## References

[1] Ruff C.T., Giugliano R.P., Braunwald E., Hoffman E.B., Deenadayalu N., Ezekowitz M.D., Camm A.J., Weitz J.I., Lewis B.S., Parkhomenko A. (2014). Comparison of the efficacy and safety of new oral anticoagulants with warfarin in patients with atrial fibrillation: A meta-analysis of randomised trials. Lancet.

[2] Connolly S.J., Ezekowitz M.D., Yusuf S., Eikelboom J., Oldgren J., Parekh A., Pogue J., Reilly P.A., Themeles E., Varrone J. (2009). Dabigatran versus warfarin in patients with atrial fibrillation. N. Engl. J. Med..

[3] Granger C.B., Alexander J.H., McMurray J.J., Lopes R.D., Hylek E.M., Hanna M., Al-Khalidi H.R., Ansell J., Atar D., Avezum A. (2011). Apixaban versus warfarin in patients with atrial fibrillation. N. Engl. J. Med..

[4] Patel M.R., Mahaffey K.W., Garg J., Pan G., Singer D.E., Hacke W., Breithardt G., Halperin J.L., Hankey G.J., Piccini J.P. (2011). Rivaroxaban versus warfarin in nonvalvular atrial fibrillation. N. Engl. J. Med..

[5] Giugliano R.P., Ruff C.T., Braunwald E., Murphy S.A., Wiviott S.D., Halperin J.L., Waldo A.L., Ezekowitz M.D., Weitz J.I., Spinar J. (2013). Edoxaban versus warfarin in patients with atrial fibrillation. N. Engl. J. Med..

[6] Heidbuchel H., Verhamme P., Alings M., Antz M., Diener H.C., Hacke W., Oldgren J., Sinnaeve P., Camm A.J., Kirchhof P. (2015). Updated European Heart Rhythm Association Practical Guide on the use of non-vitamin K antagonist anticoagulants in patients with non-valvular atrial fibrillation. EP Eur..

[7] Camm A.J., Cools F., Virdone S., Bassand J.P., Fitzmaurice D.A., Arthur Fox K.A., Goldhaber S.Z., Goto S., Haas S., Mantovani L.G. (2020). Mortality in Patients With Atrial Fibrillation Receiving Nonrecommended Doses of Direct Oral Anticoagulants. J. Am. Coll. Cardiol..

[8] Alcusky M., Tjia J., McManus D.D., Hume A.L., Fisher M., Lapane K.L. (2020). Comparative Safety and Effectiveness of Direct-Acting Oral Anticoagulants Versus Warfarin: A National Cohort Study of Nursing Home Residents. J. Gen. Intern. Med..

[9] Ruiz Ortiz M., Muniz J., Rana Miguez P., Roldan I., Marin F., Asuncion Esteve-Pastor M., Cequier A., Martinez-Selles M., Bertomeu V., Anguita M. (2018). Inappropriate doses of direct oral anticoagulants in real-world clinical practice: Prevalence and associated factors. A subanalysis of the FANTASIIA Registry. EP Eur..

[10] Arbel R., Sergienko R., Hammerman A., Greenberg-Dotan S., Batat E., Avnery O., Ellis M.H. (2019). Effectiveness and Safety of Off-Label Dose-Reduced Direct Oral Anticoagulants in Atrial Fibrillation. Am. J. Med..

[11] Huisman M.V., Rothman K.J., Paquette M., Teutsch C., Diener H.C., Dubner S.J., Halperin J.L., Ma C.S., Zint K., Elsaesser A. (2017). The Changing Landscape for Stroke Prevention in AF: Findings From the GLORIA-AF Registry Phase 2. J. Am. Coll. Cardiol..

[12] Steinberg B.A., Shrader P., Thomas L., Ansell J., Fonarow G.C., Gersh B.J., Kowey P.R., Mahaffey K.W., Naccarelli G., Reiffel J. (2016). Off-Label Dosing of Non-Vitamin K Antagonist Oral Anticoagulants and Adverse Outcomes: The ORBIT-AF II Registry. J. Am. Coll. Cardiol..

[13] Antonucci E., Poli D., Tosetto A., Pengo V., Tripodi A., Magrini N., Marongiu F., Palareti G., Register S. (2015). The Italian START-Register on Anticoagulation with Focus on Atrial Fibrillation. PLoS ONE.

[14] Cockcroft D.W., Gault M.H. (1976). Prediction of creatinine clearance from serum creatinine. Nephron.

[15] Lip G.Y., Nieuwlaat R., Pisters R., Lane D.A., Crijns H.J. (2010). Refining clinical risk stratification for predicting stroke and thromboembolism in atrial fibrillation using a novel risk factor-based approach: The euro heart survey on atrial fibrillation. Chest.

[16] Pisters R., Lane D.A., Nieuwlaat R., de Vos C.B., Crijns H.J., Lip G.Y. (2010). A novel user-friendly score (HAS-BLED) to assess 1-year risk of major bleeding in patients with atrial fibrillation: The Euro Heart Survey. Chest.

[17] Schulman S., Kearon C., Subcommittee on Control of Anticoagulation of the Scientific and Standardization Committee of the International Society on Thrombosis and Haemostasis (2005). Definition of major bleeding in clinical investigations of antihemostatic medicinal products in non-surgical patients. J. Thromb. Haemost. JTH.

[18] Kaatz S., Ahmad D., Spyropoulos A.C., Schulman S., Subcommittee on Control of Anticoagulation (2015). Definition of clinically relevant non-major bleeding in studies of anticoagulants in atrial fibrillation and venous thromboembolic disease in non-surgical patients: Communication from the SSC of the ISTH. J. Thromb. Haemost. JTH.

[19] Steinberg B.A., Shrader P., Pieper K., Thomas L., Allen L.A., Ansell J., Chan P.S., Ezekowitz M.D., Fonarow G.C., Freeman J.V. (2018). Frequency and Outcomes of Reduced Dose Non-Vitamin K Antagonist Anticoagulants: Results from ORBIT-AF II (The Outcomes Registry for Better Informed Treatment of Atrial Fibrillation II). J. Am. Heart Assoc..

[20] Santos J., Antonio N., Rocha M., Fortuna A. (2020). Impact of direct oral anticoagulant off-label doses on clinical outcomes of atrial fibrillation patients: A systematic review. Br. J. Clin. Pharmacol..

[21] Gibson C.M., Smith C.B., Davis S., Scalese M.J. (2018). Assessment of Apixaban Prescribing Patterns for Nonvalvular Atrial Fibrillation in Hospitalized Patients. Ann. Pharmacother..

[22] Bo M., Corsini A., Brunetti E., Isaia G., Gibello M., Ferri N., Poli D., Marchionni N., De Ferrari G.M. (2021). Off-label use of reduced dose direct oral factor Xa inhibitors in subjects with atrial fibrillation: A review of clinical evidence. Eur. Heart J. Cardiovasc. Pharmacother..

[23] Bassand J.P., Virdone S., Badoz M., Verheugt F.W.A., Camm A.J., Cools F., Fox K.A.A., Goldhaber S.Z., Goto S., Haas S. (2021). Bleeding and related mortality with NOACs and VKAs in newly diagnosed atrial fibrillation: Results from the GARFIELD-AF registry. Blood Adv..

[24] Kirchhof P., Ammentorp B., Darius H., De Caterina R., Le Heuzey J.Y., Schilling R.J., Schmitt J., Zamorano J.L. (2014). Management of atrial fibrillation in seven European countries after the publication of the 2010 ESC Guidelines on atrial fibrillation: Primary results of the PREvention oF thromboemolic events—European Registry in Atrial Fibrillation (PREFER in AF). EP Eur..

[25] Caso V., de Groot J.R., Sanmartin Fernandez M., Segura T., Blomstrom-Lundqvist C., Hargroves D., Antoniou S., Williams H., Worsley A., Harris J. (2023). Outcomes and drivers of inappropriate dosing of non-vitamin K antagonist oral anticoagulants (NOACs) in patients with atrial fibrillation: A systematic review and meta-analysis. Heart.

[26] Farjat A.E., Virdone S., Thomas L.E., Kakkar A.K., Pieper K.S., Piccini J.P. (2022). The importance of the design of observational studies in comparative effectiveness research: Lessons from the GARFIELD-AF and ORBIT-AF registries. Am. Heart J..

